# Decrypting Cryptogenic Hepatocellular Carcinoma: Clinical Manifestations, Prognostic Factors and Long-Term Survival by Propensity Score Model

**DOI:** 10.1371/journal.pone.0089373

**Published:** 2014-02-24

**Authors:** Chia-Yang Hsu, Yun-Hsuan Lee, Po-Hong Liu, Cheng-Yuan Hsia, Yi-Hsiang Huang, Han-Chieh Lin, Yi-You Chiou, Fa-Yauh Lee, Teh-Ia Huo

**Affiliations:** 1 Faculty of Medicine, National Yang-Ming University School of Medicine, Taipei, Taiwan; 2 Institute of Clinical Medicine, National Yang-Ming University School of Medicine, Taipei, Taiwan; 3 Institute of Pharmacology, National Yang-Ming University School of Medicine, Taipei, Taiwan; 4 Department of Medicine, Taipei Veterans General Hospital, Taipei, Taiwan; 5 Department of Surgery, Taipei Veterans General Hospital, Taipei, Taiwan; 6 Department of Radiology, Taipei Veterans General Hospital, Taipei, Taiwan; 7 Department of Biostatistics, UCLA, Los Angeles, California, United States of America; CRCL-INSERM, France

## Abstract

**Background and Aims:**

The clinical aspects of cryptogenic hepatocellular carcinoma (HCC), defined as HCC in patients without hepatitis B, C or alcoholism, are not clear. We investigated its clinical presentations, long-term survival and prognostic predictors.

**Methods:**

A total of 2645 HCC patients were studied. One-to-one matched pairs between viral/alcoholic and cryptogenic HCC patients were generated by using the propensity model. The survival analysis was performed with the Kaplan-Meier method and log-rank test, and hazard ratios were calculated with Cox proportional hazards model.

**Results:**

Among 366 (14%) patients with cryptogenic HCC, 34% of patients were presented with abdominal discomfort, and 31% of patients were identified incidentally. Compared to patients with viral/alcoholic HCC, cryptogenic HCC patients were significantly older (p<0.0001), with poorer performance status (p = 0.0031) and less often underwent curative treatment (p = 0.0041). They also had larger tumor burden (p<0.0001), poorer renal function (p<0.0001), lower α-fetoprotein level (p<0.0001), and more advanced Barcelona Clinic Liver Cancer stages (p<0.0001). With propensity score model, 366 pairs of similar HCC patients were selected and similar long-term survival between the two groups of patients was found (p = 0.1038). For cryptogenic HCC patients, α-fetoprotein ≧49 ng/mL (hazard ratio [HR]: 1.955, p = 0.0002), Child-Turcotte-Pugh class B/C (HR: 2.798, p<0.0001), performance status ≧1 (HR: 2.463, p<0.0001) and vascular invasion (HR: 1.608, p = 0.0257) were independent predictors of poor prognosis.

**Conclusions:**

Patients with cryptogenic HCC are usually diagnosed with poor general condition at late stages. However, cryptogenic HCC patients have similar prognostic predictors and long-term survival compared with viral/alcoholic HCC patients. Diagnosis at an early stage may improve their clinical outcomes.

## Introduction

Hepatocellular carcinoma (HCC) is one of the most common malignant neoplasms worldwide [1]. For patients diagnosed at early stages, curative treatments including surgical resection, percutaneous ablation and transplantation provide the chance of long-term remission with 5-year survival rate of up to 75% [1]. Unfortunately, for symptomatic patients, the majority of them are not suitable to undergo aggressive treatments because of poor hepatic reserve or overwhelming tumor burden. Most of them receive palliative treatments such as transarterial chemoembolization (TACE), targeted therapy and best palliative management to prolong survival or improve the quality of life [2], [3].

Hepatitis B, hepatitis C and alcoholic hepatitis are common chronic liver diseases which could result in liver cirrhosis and HCC. Hepatitis B is the major risk factor of HCC in Asia and Africa, whereas hepatitis C is the predominant etiology in Japan and Western countries [4]. In the past decade, the importance of nonalcoholic steatohepatitis (NASH) related cirrhosis or HCC has increased gradually due to the obese population in developed countries [5]. However, there is still a substantial portion of HCC patients without definite history of chronic liver disease at the time of diagnosis [6], [7]. In countries with high prevalence rate of hepatitis B or C, prevention programs such as hepatitis B vaccination and regular follow-up for chronic liver diseases have been carried out in order to reduce the incidence of HCC [8], [9]. As for patients with non-viral and non-alcoholic HCC, there are few studies addressing the adequate surveillance strategy, clinical features or long-term prognosis, and most of them are diagnosed at advanced stages when symptoms surface.

Cryptogenic HCC, defined as non-viral, non-alcoholic HCC and no other known etiology of chronic liver disease, has been seldom systemically studied. In addition, most conclusions were obtained from small HCC cohort and the long-term survival of these patients was not fully examined [10], [11]. In this study, we have investigated its clinical presentations, demographic characteristics, and predictors of long-term outcome in a prospectively collected cohort; the survival distributions of patients with cryptogenic or viral/alcoholic HCC were compared in the propensity model.

## Patients and Methods

### Patients

A prospective database of patients with HCC collected during an 11-year period from 2002 to 2013 at Taipei Veterans General Hospital, Taiwan, formed the basis of this study. A total of 2645 treatment-naïve HCC patients were identified. The baseline information, including patient characteristics, causes of chronic liver disease, serum biochemistry, severity of cirrhosis, performance status and cancer stages, were recorded when the diagnosis was made. Part of the study patients had been enrolled in our previous studies [12], [13]. This study has been approved by the institutional review board of Taipei Veterans General Hospital, Taiwan, and complies with the standards of Declaration of Helsinki and current ethical guidelines. All medical records were de-identified prior to analysis.

### Diagnosis and Definitions

The diagnosis of HCC was based on the findings of typical radiological characteristics in at least two imaging modalities including ultrasound, hepatic arterial angiography, magnetic resonance (MR) imaging and contrast-enhanced dynamic computed tomography (CT); or histologically confirmed or by a single positive imaging technique accompanied with serum α-fetoprotein (AFP) level >400 ng/mL [14], [15]. Alcoholism was diagnosed in patients with daily consumption of at least 40 g of alcohol for 5 years or more [16]. Patients were considered to have hepatitis C virus (HCV) infection if they were seropositive for antibody against HCV (anti-HCV) by a second-generation enzyme immunoassay (Abbott Laboratories). Hepatitis B virus (HBV) infection was diagnosed if hepatitis B surface antigen (HBsAg) was found serologically (RIA kits, Abbott Laboratories). Ascites defined as free peritoneal fluid was discovered by abdominal sonography, CT or MR imaging [17]. Performance status was determined at entry according to the Eastern Cooperative Oncology Group (ECOG) criteria [18].

The estimated glomerular filtration rate (eGFR) was calculated by using the modification of diet in renal disease formula [19]. Vascular invasion was diagnosed by contrast-enhanced imaging modalities. Total tumor volume was calculated according to mathematical equations as described in our previous studies [20].

### Treatment

Surgical resection, transplantation and percutaneous ablation (acetic or ethanol acid injection and radiofrequency ablation) were collectively classified as curative treatment in this study [21]. TACE, targeted therapy, systemic chemotherapy and best supportive care were classified as non-curative treatment [22].

### Propensity Score Analysis

To compare the overall survival between patients with viral/alcoholic and cryptogenic HCC in a prospectively collected cohort, a propensity score model and greedy algorithm matching without replacement were used with an attempt to reduce potential biases in survival analysis [23], [24]. Possible variables associated with long-term survival of HCC patients, including age, sex, tumor burden, severity of cirrhosis, performance status, vascular invasion, renal function, AFP, curative treatments, the Barcelona-Clinic Liver Cancer (BCLC) and Cancer of the Liver Italian Program (CLIP) staging systems were included comprehensively for propensity score generation. With these selected variables, a logistic regression was applied to generate a continuous propensity score from 0 to 1. One-to-one matches between patients with viral/alcoholic and cryptogenic HCC were introduced into the subsequent analysis.

### Statistics

Categorical data were compared with chi-squared test between patients with viral/alcoholic and cryptogenic HCC. Continuous demographic characteristics were compared with Mann-Whitney ranked sum test. The comparison of survival distribution was performed by the Kaplan-Meier method with a log-rank test. For prognostic predictor analysis, continuous variables were split by the median or clinically meaningful cut-off values and treated as dichotomous covariates. Each possible prognostic predictor was tested by the Kaplan-Meier method initially; and factors which were significant in the univariate analysis were introduced into the multivariate Cox proportional hazards model to calculate the adjusted hazard ratios. A p value was considered statistically significant when it was less than 0.05. All statistical analyses were conducted with the SAS 9.0 (SAS institute, North Carolina).

## Results

### Patients Characteristics

Patients were classified as cryptogenic HCC if there was no evidence of hepatitis B, hepatitis C or alcoholism-related HCC. In addition, 10 patients with known etiologies of chronic liver disease such as primary biliary cirrhosis, hemochromatosis, and Wilson’s disease were excluded. A total of 366 (14%) cryptogenic HCC patients were identified. As shown in Table 1, cryptogenic HCC patients were significantly older (p<0.0001), with poorer performance status (p = 0.0031) and poorer renal function (p<0.0001). They also had significantly larger tumor size and total tumor volume (both p<0.0001), advanced BCLC stages (p<0.0001) and more often to receive non-curative therapy (p = 0.0041).

**Table 1:** Comparison of demographic characteristics between patients with cryptogenic HCC and viral/alcoholic HCC.

Variables	Viral and alcoholic HCC (n = 2269)	Cryptogenic HCC (n = 366)	P
Age (years, mean ± SD)	63±13	72±13	<0.0001
Male (%)	1757 (77)	283 (77)	0.9619
Chronic liver disease			
Positive HBsAg (%)	1456 (64)	0	
Positive anti-HCV (%)	810 (36)	0	
HBV+HCV (%)	116 (5)	0	
Alcoholism (%)	447 (20)	0	
CTP class A (%)	1641 (72)	263 (72)	0.8538
Ascites (%)	554 (24)	92 (25)	0.7662
Tumor size ≧ 3 cm (%)	1496 (67)	289 (79)	<0.0001
Multiple tumors (%)	929 (41)	121 (33)	0.0043
Tumor volume (cm^3^, median)	357±711 (44)	466±793 (144)	<0.0001
Performance status 0 (%)	1340 (59)	186 (51)	0.0031
Vascular invasion (%)	655 (29)	105 (29)	0.9441
Biochemistry (mean ± SD)			
Albumin (g/dL)	3.7±0.6	3.7±0.7	0.1617
Bilirubin (mg/dL)	1.6±2.8	1.3±2.2	<0.0001
Creatinine (mg/dL)	1.2±1.1	1.3±0.8	<0.0001
INR of PT	1.09±0.2	1.05±0.2	<0.0001
Sodium (mmol/L)	138±3.9	138±4.1	0.7036
Estimated GFR (mL/min/1.73 m^2^)	77±32 (75)	66±28 (64)	<0.0001
AFP (ng/mL, median)	26,823±252,893 (57)	20,958±158,676 (14)	<0.0001
BCLC stage 0/A/B/C/D (%)	6/23/13/44/14	4/16/16/44/21	<0.0001
CLIP score 0/1/2/3/4/5/6 (%)	27/25/16/12/12/7/2	28/22/14/17/10/7/2	0.1225
Curative treatments (%)	1057 (47)	141 (39)	0.0041

AFP, α-fetoprotein; BCLC, Barcelona Clinic Liver Cancer; CLIP, Cancer of the Liver Italian Program; CTP, Child-Turcotte-Pugh; GFR, glomerular filtration rate; HBV, hepatitis B virus; HCV, hepatitis C virus; INR, international ratio; PT, prothrombin time; SD, standard deviation.

Curative treatments include resection, radiofrequency ablation, percutaneous alcohol/acetic acid injection, and transplantation.

For patients with viral/alcoholic HCC, they had more tumor nodules (p = 0.0043), higher serum bilirubin and AFP levels (both p<0.0001) and longer prothrombin time (p<0.0001). Otherwise there were no significant differences in gender (p = 0.9619), Child-Turcotte-Pugh (CTP) classification (p = 0.8538), prevalence of ascites and vascular invasion (p = 0.7662 and 0.9441), serum levels of albumin and sodium (p = 0.1617 and 0.7036) and the CLIP score (p = 0.1225) between viral/alcoholic and cryptogenic HCC patients.

### Primary Presentations

As indicated in Table 2, one-third (34%) of cryptogenic HCC patients were presented to the hospital due to abdominal discomfort, including dull or sharp pain, and fullness sensation over upper abdomen. The second most common presentation was incidental finding (31%), which was so defined that the diagnosis of HCC was made during medical management not related to liver disease, or physical check-up for asymptomatic patients without a prior history of liver disease. Systematic presentations including poor appetite (6%), general weakness (6%), body weight loss (3%) and fever (2%) were noted at the time of diagnosis. There were only 21 patients (6%) under regular follow-up programs for non-viral/alcoholic hepatitis related cirrhosis when HCC was diagnosed.

**Table 2:** Primary complaint or presentation at diagnosis of patients with cryptogenic HCC.

Cryptogenic HCC patients (n = 366)	Number (%)
Abdominal discomfort	125 (34)
Incidental finding	112 (31)
Poor appetite	22 (6)
Non-viral, non-alcoholic cirrhosis underregular surveillance	21 (6)
General weakness	21 (6)
Elevated liver enzymes	20 (6)
Body weight loss	10 (3)
Yellowish skin/tea-color urine	8 (2)
Fever	8 (2)
Gastrointestinal bleeding	7 (2)
Lower leg edema	5 (1)
Palpable abdominal mass	4 (1)
Nausea and/or vomiting	3 (1)

### Survival Comparison Between Viral/alcoholic and Cryptogenic HCC Patients

**Figure 1:**
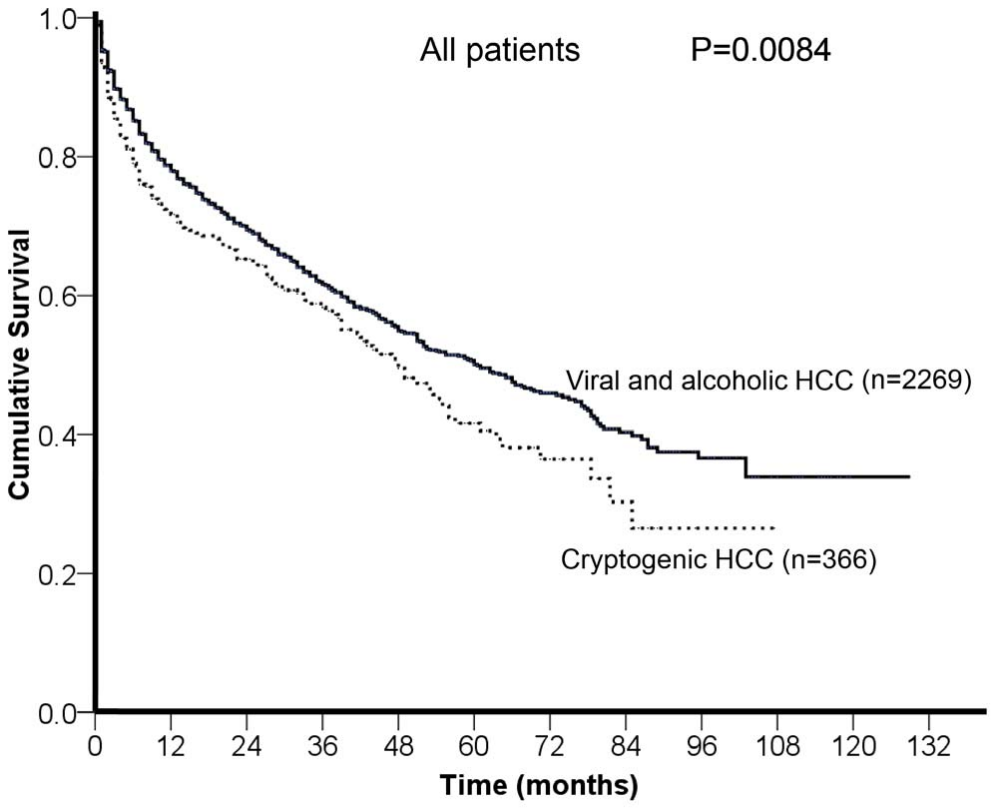
Comparison of the survival distribution between patients with cryptogenic HCC and viral/alcoholic HCC. Cryptogenic HCC patients had a significantly worse survival than viral/alcoholic HCC patients (p = 0.0084).

The comparison of long-term survival between viral/alcoholic and cryptogenic HCC patients was given in Fig. 1. During a mean follow-up period of 27±26 months, cryptogenic HCC patients had a significantly shorter survival than did patients with viral/alcoholic etiologies (p = 0.0084).

### Characteristics of Patients Selected in the Propensity Model

By using the propensity model, 366 pairs of matched HCC patients were selected (Table 3). Among matched patients stratified by the etiology of HCC, there were no significant differences in age, sex, severity of cirrhosis, tumor burden, performance status, prevalence of ascites and vascular invasion, renal function, serum AFP level, treatment modalities and the scores of BCLC and CLIP systems (all p>0.05).

**Table 3:** Comparison of demographic characteristics between patients with cryptogenic HCC and viral/alcoholic HCC in the propensity model.

Variables	Viral and alcoholic HCC (n = 366)	Cryptogenic HCC (n = 366)	P
Age ≧ 65 years (%)	273 (75)	273 (75)	1
Male (%)	284 (78)	283 (77)	0.9295
CTP class A (%)	249 (68)	263 (72)	0.2591
Ascites (%)	92 (25)	92 (25)	1
Tumor size ≧ 3 cm (%)	273 (75)	289 (79)	0.1614
Multiple tumors (%)	118 (32)	121 (33)	0.8131
Tumor volume ≧ 50.9 cm^3^	231 (63)	236 (64)	0.7006
Performance status 0 (%)	201 (55)	186 (51)	0.2667
Vascular invasion (%)	114 (31)	105 (29)	0.4676
Estimated GFR ≧ 60 mL/min/1.73 m^2^	214 (58)	214 (58)	1
AFP ≧ 49 ng/mL	141 (39)	141 (39)	1
BCLC stage 0/A/B/C/D (%)	6/17/17/45/15	4/16/16/44/21	0.2185
CLIP score 0/1/2/3/4/5/6 (%)	28/25/15/12/12/7/1	28/22/14/17/10/7/2	0.5112
Curative treatments (%)	147 (40)	141 (39)	0.6499

### Survival Analysis in the Propensity Model

**Figure 2:**
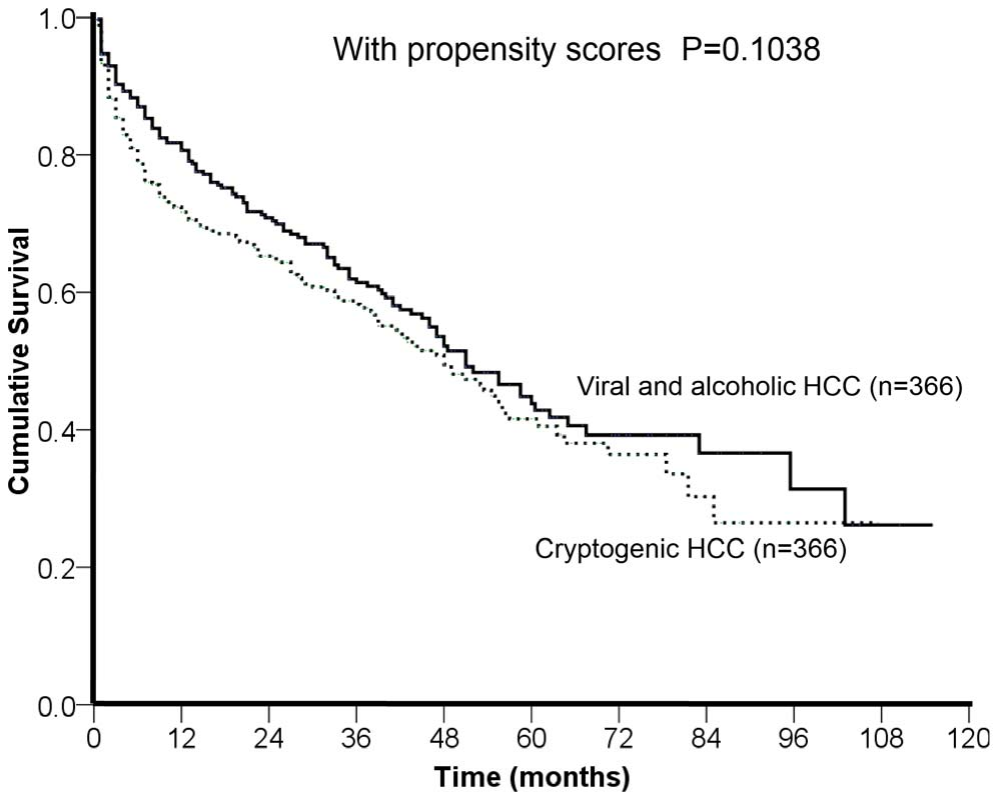
Comparison of the survival distribution of cryptogenic and viral/alcoholic HCC patients in the propensity model. There was no significant difference of overall survival between patients with cryptogenic and viral/alcoholic HCC (p = 0.1038).

During a mean follow-up period of 26±25 months, the comparison of long-term survival between patients with viral/alcoholic and cryptogenic HCC showed no significant difference in the propensity model (p = 0.1038; Fig. 2).

### Predictors of Long-term Survival in Cryptogenic HCC Patients

Potential prognostic predictors including age, gender, number and size of tumor nodules, total tumor volume, CTP class, presence of ascites and vascular invasion, serum AFP level, performance status, renal function, and treatment modalities were examined by using the Kaplan-Meier analysis and log-rank test. Factors associated with decreased overall survival were older age (p = 0.0159), advanced CTP classification (p<0.0001), ascites (p<0.0001), larger tumor size (p = 0.0007), multiple tumors (p = 0.0312), larger tumor volume (p<0.0001), poorer performance status (p<0.0001), vascular invasion (p<0.0001), poorer renal function (p = 0.0005), higher AFP level (p<0.0001), and non-curative treatments (p<0.0001). When these variable were introduced into the Cox proportional hazards model to generate adjusted hazard ratios, four predictive factors, including serum AFP level ≧49 ng/mL (hazard ratio [HR]: 1.955, 95% confidence interval [CI]: 1.371–2.787, p = 0.0002), CTP class B or C (HR: 2.798, 95% CI: 1.694–4.621, p<0.0001), performance status ≧1 (HR: 2.463, 95% CI: 1.640–3.697, p<0.0001), and vascular invasion (HR: 1.608, 95% CI: 1.059–2.44, p = 0.0257), were identified as independent predictors of poor prognosis (Table 4).

**Table 4:** Independent predictors of poor prognosis in patients with cryptogenic HCC in Cox proportional hazard model.

	Hazard ratio	95% CI	P
Cryptogenic HCC (n = 366)			
AFP ≧ 49 ng/mL	1.955	1.371–2.787	0.0002
CTP class B or C	2.798	1.694–4.621	<0.0001
Performance status ≧ 1	2.463	1.64–3.697	<0.0001
Vascular invasion	1.608	1.059–2.44	0.0257

CI, confidence interval.

## Discussion

Hepatitis B, hepatitis C and alcoholism are considered three major etiologies of chronic liver diseases that could subsequently result in cirrhosis or HCC. Genetic liver disease, autoimmune liver disease and NASH are also associated with chronic liver injury and various complications. In general, cryptogenic HCC is used to describe HCC in patients without viral/alcoholic hepatitis after other known etiologies such as autoimmune, genetic, toxin-related liver diseases, NASH, Wilson’s disease and hemochromatosis are excluded. The prevalence rate, associated factors, and distribution of long-term survival of cryptogenic HCC were not fully investigated due to insufficient case number and variable definitions from different research groups [7], [25]. In this study, cryptogenic HCC was diagnosed in 14% of HCC patients from a large prospective patient cohort. Cryptogenic HCC patients usually had poor general condition and larger tumor(s) at the time of diagnosis. After potential biases caused by different baseline characteristics were removed in the propensity model, we found that there was no significant difference of long-term survival between cryptogenic and viral/alcoholic HCC patients. Our results indicate that early diagnosis with effective surveillance program may enhance the survival in these patients.

Small HCC(s) seldom causes clinical symptoms, therefore it is often very difficult to diagnose small HCC(s) in patients without a prior history of liver disease. When clinical symptoms develop, HCC patients are usually presented with large tumor burden and poor general condition. Hence, most of them could only receive non-curative treatments and are associated with a less favorable prognosis [26], [27]. In our study, nearly two-thirds of patients with cryptogenic HCC were diagnosed when clinical manifestations were apparent. About 17% of patients presented with systemic symptoms such as poor appetite, weakness, body weight loss and fever. For these patients, it is more challenging for physicians to reach a definite diagnosis. Only 6% of cryptogenic HCC patients were diagnosed under regular chronic liver disease follow-up program. In comparison to patients with viral/alcoholic HCC, who received regular follow-up after the diagnosis of viral hepatitis or alcoholic liver disease was made, patients with cryptogenic HCC often do not receive regular follow-up until symptomatic cirrhosis develop. Advanced cirrhosis *per se* could preclude the possibility of patients with cryptogenic HCC to receive aggressive treatments due to the status of liver decompensation. Taken together, the majority of cryptogenic HCC patients did not receive comprehensive liver studies until symptoms of HCC developed. This could result in delayed diagnosis, limited choice of treatment and poor prognosis.

Comparison of baseline characteristics between viral/alcoholic and cryptogenic HCC (Table 1) highlighted the different compositions in these two groups. Patients with cryptogenic HCC were significantly older and had poorer performance status and renal function; in addition, cryptogenic HCC patients had significantly larger tumor size and total tumor volume. Not surprisingly, cryptogenic HCC patients had more advanced BCLC stages that make curative therapy less likely to perform. Importantly, the analysis of overall survival might be confounded by these crucial baseline differences (Fig. 1). In this study, propensity score analysis with one-to-one nearest neighbor matching selected 366 pairs of similar patients (Table 3). After baseline confounders were removed, the survival analysis showed comparable long-term survival distribution. There findings not only show the evidence of confounding effects caused by larger tumor burden and poor general condition, but also suggest that cryptogenic HCC patients might have an improved prognosis if the diagnosis of HCC could be made earlier.

Patients with viral/alcoholic cirrhosis had significantly higher AFP level, which might be associated with ongoing hepatitis at the time of diagnosis [28], [29]; they also had significantly higher serum bilirubin and longer prothrombin time in comparison with cryptogenic HCC patients. However, these differences were not large enough to make an impact on CTP classification. It is noteworthy that more viral/alcoholic HCC patients had multiple tumors in our study. This is possibly because that liver parenchyma in patients with viral/alcoholic HCC suffered from chronic liver injury for decades, and the whole liver may become fertile soil for the development of HCC nodule(s).

Non-alcoholic fatty liver disease (NAFLD), including NASH, has been increasingly considered an underlying cause of HCC [25], [30]. However, several reasons make NAFLD-related HCC a difficult-to-diagnose clinical entity: (1) for patients with viral hepatitis and possible simultaneous NAFLD, usually they were classified as viral hepatitis-related HCC, (2) for patients with chronic liver disease, cirrhosis or HCC, most of them did not receive liver biopsy on a routine basis, and (3) with the progression of liver injury, the typical pathologic features of NAFLD/NASH may disappear when cirrhosis develops. For our HCC cohort, which enrolled every patient when the diagnosis of HCC was made, it was difficult to evaluate the presence of NAFLD/NASH even with biopsied or resected liver tissue [31]. Hence, we did not specifically exclude NAFLD-related HCC from cryptogenic HCC in this study, and further studies are required to delineate the role of NASH in cryptogenic HCC.

The prognostic predictors of patients with cryptogenic HCC had not been comprehensively studied due to very limited case number in published literatures. In this study, we have determined the predictors of long-term survival by using a large, prospectively enrolled HCC cohort. High serum AFP level and vascular invasion, which are both associated with large tumor burden, are identified as independent predictors of poor outcome. The association between advanced CTP class and poor survival further discloses the importance of hepatic reserve. In addition, consistent with published series, the multivariate Cox model shows that poor performance status was a strong predictor of decreased long-term survival [18]. These findings suggest that in spite of different underlying etiologies, cryptogenic HCC shares similar prognostic predictors with patients with viral/alcoholic HCC [32], [33].

There are a few potential limitations of this study. Firstly, in this study, more than half of our patients had evidence of hepatitis B infection. This feature is distinctly different from countries where hepatitis C infection and NAFLD were the predominant etiologies of chronic liver disease [34]. Secondly, patients with hepatitis B or C might have received anti-viral treatment to suppress viral activity some time during the follow-up period, and this may alter the prognosis [35], [36].

In conclusion, our results indicate that cryptogenic HCC are often diagnosed at late stages in older patients. However, cryptogenic HCC is not associated with a poor prognosis after confounding effects are abolished. For these patients, early diagnosis could substantially improve their long-term survival. Our findings also imply that a subset of HCC patients might have a chance to benefit from adequate surveillance program. The optimal surveillance guidelines for this particular patient group need further studies to establish.
